# Supplementary material for: Polydopamine-Modified Al2O3/Polyurethane Composites with Largely Improved Thermal and Mechanical Properties
Source: Materials (Basel). 2020 Apr 9;13(7):1772. doi: 10.3390/ma13071772 (PMC7179027; doi:10.3390/ma13071772)
Supplement: Supplementary file 1 [file materials-13-01772-s001.pdf]

*materials*

*Article*

# Polydopamine-Modified $Al_2O_3$/Polyurethane Composites with Largely Improved Thermal and Mechanical Properties

**Ruikui Du [1,2,*], Li He [1,2], Peng Li [1,2], Guizhe Zhao [1,2,*]**

[1] North University of China, Taiyuan 030051, China; zminggu@163.com (L.H.); lpzbdx@163.com (P.L.)
[2] Shanxi Province Polymer Composite Engineering Technology Research Center, Taiyuan 030051, China
* Correspondence: dukui1107@163.com (R.D.); zgz@nuc.edu.cn (G.Z.)

Received: 15 February 2020; Accepted: 6 April 2020; Published: date

We can find that unmodified alumina is exposed to the fracture surface, which indicates poor interface adhesion between $Al_2O_3$ and pure PU. And there are some serious agglomerations. Polydopamine-modified composites have better dispersion. When the particle content is small, alumina is surrounded by PU in the composites, and the particles cannot contact each other well, failing to form a good thermal conduction path. Therefore, when the particle content is low, the thermal conductivity is also poor, which is consistent with the results of the above thermal conductivity.

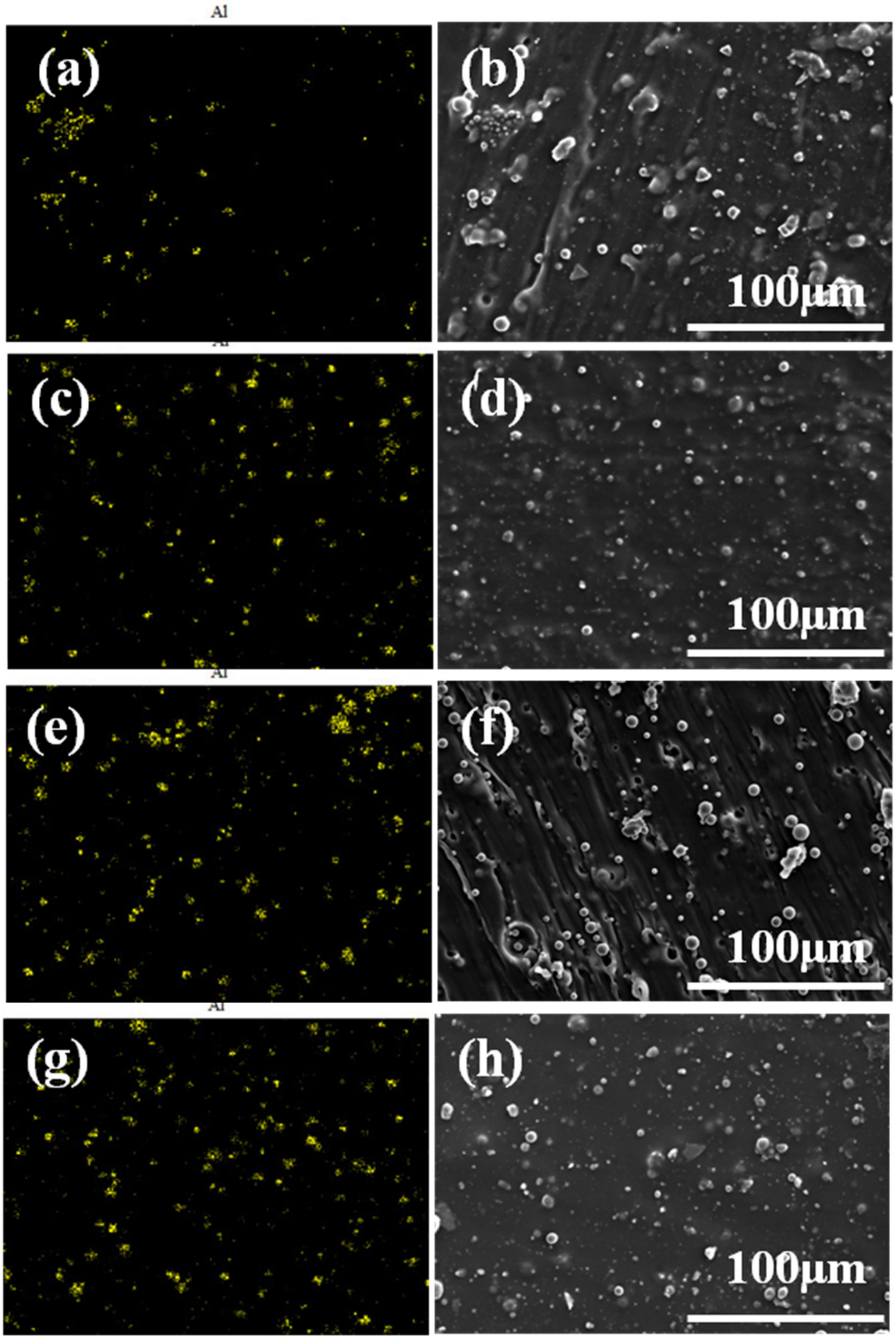


**Figure S1.** SEM morphology of $Al_2O_3$/PUand PDA-$Al_2O_3$/PU composites filled with (a) 10 wt% $Al_2O_3$, (c) 10 wt% PDA-$Al_2O_3$, (e) 20 wt% $Al_2O_3$, (g) 20 wt% PDA-$Al_2O_3$, EDS element distribution of yellow spots in (b) 10 wt% $Al_2O_3$/PU (d) 10 wt% PDA-$Al_2O_3$/PU (f) 20 wt% $Al_2O_3$/PU and (h) 20 wt% PDA-$Al_2O_3$/PU.

© 2020 by the authors. Submitted for possible open access publication under the terms and conditions of the Creative Commons Attribution (CC BY) license (http://creativecommons.org/licenses/by/4.0/).
